# Towards Verifying the Imported Soybeans of China Using Stable Isotope and Elemental Analysis Coupled with Chemometrics

**DOI:** 10.3390/foods12234227

**Published:** 2023-11-23

**Authors:** Xiuwen Zhou, Beibei Xiong, Xiao Ma, Baohui Jin, Liqi Xie, Karyne M. Rogers, Hui Zhang, Hao Wu

**Affiliations:** 1 Department of Ocean Science and Engineering, Southern University of Science and Technology, Shenzhen 518055, China; 2 Food Inspection and Quarantine Center, Shenzhen Customs, Shenzhen 518033, China; 3 Department of Chromatography and Mass Spectrometry, Thermo Fisher Scientific (China) Co., Ltd., Shanghai 201206, China; 4 National Isotope Centre, GNS Science, Lower Hutt 5040, New Zealand; 5 Comprehensive Technology Centre, Zhangjiagang Customs, Suzhou 215000, China; 6 Key Laboratory of the Ministry of Education for Coastal and Wetland Ecosystems, College of the Environment and Ecology, Xiamen University, Xiamen 361102, China

**Keywords:** trace elements, stable isotopes, soybean, geographical origin, chemometrics, traceability, China

## Abstract

Verifying the geographical origin of soybeans (*Glycine max* [Linn.] Merr.) is a major challenge as there is little available information regarding non-parametric statistical origin approaches for Chinese domestic and imported soybeans. Commercially procured soybean samples from China (*n* = 33) and soybeans imported from Brazil (*n* = 90), the United States of America (*n* = 6), and Argentina (*n* = 27) were collected to characterize different producing origins using stable isotopes (*δ*^2^H, *δ*^18^O, *δ*^15^N, *δ*^13^C, and *δ*^34^S), non-metallic element content (% N, % C, and % S), and 23 mineral elements. Chemometric techniques such as principal component analysis (PCA), linear discriminant analysis (LDA), and BP–artificial neural network (BP-ANN) were applied to classify each origin profile. The feasibility of stable isotopes and elemental analysis combined with chemometrics as a discrimination tool to determine the geographical origin of soybeans was evaluated, and origin traceability models were developed. A PCA model indicated that origin discriminant separation was possible between the four soybean origins. Soybean mineral element content was found to be more indicative of origin than stable isotopes or non-metallic element contents. A comparison of two chemometric discriminant models, LDA and BP-ANN, showed both achieved an overall accuracy of 100% for testing and training sets when using a combined isotope and elemental approach. Our findings elucidate the importance of a combined approach in developing a reliable origin labeling method for domestic and imported soybeans in China.

## 1. Introduction

Cultivated soybean (*Glycine max* [Linn.] Merr.) evolved from wild soybean (*G. soja* Sieb. & Zucc.) and originated in China around 5000 years ago [1]. Recognized as one of the foremost contributors to sources of oil, food, traditional Chinese medicine, and feed crops, soybeans also play a vital role in various industrial applications, particularly in biodiesel fuel production [2]. Soybean contains about 18% oil and 38% protein, which constitutes 60% of the global vegetable protein provision [3], and it has become one of the most economically important oilseed and biodiesel crops. China’s importance as a world soybean producer has steadily declined in the last decade despite its domestication and continued high consumption [4]. According to data from the General Administration of Customs of China, China is now the world’s largest importer of soybeans. In 2020, China imported a total of 100.33 million tons of soybeans, which is the highest since 2016, yet produced only 19.6 million tons of soybeans that year. The total import of soybeans into China declined to 96.52 million tons in 2021 and 91.081 million tons in 2022, which was a decrease of 5.6% compared to 2021 [5]. This was mainly due to the rise of global soybean prices and the reluctance of the United States of America (USA) to export soybeans. In terms of soybean exporting countries, in 2022, Brazil replaced the USA as the top exporter, accounting for 59.73% of the export market, followed by the USA, accounting for 32.42%, and Argentina, accounting for 4.01%. The remaining 1.88% of soybean exports mainly come from countries such as Russia, Canada, Ukraine, Ethiopia, and Tanzania. Differences in growth environment, cultivar variety, and agricultural practices can all lead to varying quality parameters of soybeans. Moreover, different processing conditions such as moisture, temperature, and drying time lead to different nutritional components and quality parameters of soybeans [6]. Compared with large-scale western mechanized planting, soybean cultivation costs are higher in China due to higher land cost or rental and labor charges, which lowers the cost competitiveness of Chinese soybean and increases its price in the open market. Soybeans are highly monitored crops due to China’s food security policies, yet because of different soybean trade policies, the quality and price of imported soybeans vary considerably. Therefore, developing soybean origin traceability technology is of high importance for protecting China’s soybean import trade and maintaining safety standards for imported soybeans.

Food fraud and adulteration have increased significantly in recent times, motivated by rapid economic gains. The consumption of labeled, branded, and certified food has become a major trend driven by changes in consumer attitudes towards nutritional health and food safety [7]. In 1992, the European Union (EU) proposed that products from specific geographical origins should be granted Geographical Indications (PGI) [8]. This initiative enables consumers to identify quality products, while producers use the label as an added marketing tool to increase crop value [9]. In China, about 40 percent of its soybeans are cultivated in Heilongjiang Province [10], which is widely recognized as the highest-quality product and has been authorized as a PGI product in China. Origin mislabeling leads to increased profits for sellers and increases the risk of fraud for consumers. PGI protection through geographical origin verification tools is now essential for all at-risk products. Metabolomic studies aim to study differences among food products and predict class memberships using statistical models, which are primely discriminative and predictive. Therefore, obtaining food fingerprint information through appropriate technologies can ensure quality and safety and fulfill origin identification purposes [11].

Methods used to determine food geographical origins, such as stable isotopes and elemental content analysis, have been recognized as particularly useful origin discriminators as the elemental composition and stable isotope signatures (*δ*^2^H, *δ*^18^O, *δ*^15^N, *δ*^13^C, and *δ*^34^S) of plants reflect their growing environment, climate, and human interactions. Different photosynthetic pathways of plants and their cultivars have different biological effects on carbon dioxide respiration processes and uptake. These differences in plant tissue ^13^C/^12^C ratios are the main determinant of bulk *δ*^13^C signatures [12]. The δ^15^N values of naturally growing plants are mainly related to plant organs, plant age, climatic conditions, biological N_2_ fixation, and soil properties, and they are also susceptible to nitrogen fertilization [13]. Plant δ^34^S values are influenced by soil elements (e.g., synthetic SO_4_^2−^ fertilizers, gypsum, and elemental sulfur S_o_), atmospheric deposition (e.g., rainwater and industrial pollution), and sea spray in some coastal regions [14,15]. Other factors influencing *δ*^34^S values include soil sulfate reduction by anaerobic bacteria on a range of diffuse sources [16]. Stable isotope values of hydrogen and oxygen are basically used to determine provenance, and these values are the results of isotopic fractionation produced during the meteorological water cycle that constitutes the evaporation, condensation, and precipitation of groundwater and shows systematic geographic isotopic variation [9,17]. Hydrogen and oxygen isotope values in plants are determined by precipitation or irrigation water effects, such as season, local climate, and geographical factors (e.g., latitude, altitude, and distance from the sea) [18]. Previous multi-element analysis established that the mineral element compositions of organisms are directly affected by regional climatic conditions, biological and environmental interactions, and metabolism [19]. Soil conditions (e.g., soil type, mineral content, pH, porosity, and humus content) are the main factors affecting the mineral content of plants [20]. ICP-MS (Inductively Coupled Plasma Mass Spectrometry) has the advantages of high sensitivity and short-time detection and is suitable for the simultaneous analysis of multiple mineral elements [19].

Research endeavors have been conducted to ascertain the provenance and traceability of agricultural products using stable isotopes and elements combined with chemometrics: Cassiae Semen tea [21], Chinese garlic [18], Trachinotus ovatus and Pampus argenteus [22], avocado [9], durian [23], rice [24], apple juice concentrates [25], wine [26,27], and Mediterranean mussels [28]. Recently, Tanaka et al. [29] used neodymium isotope ratio (^143^Nd/^144^Nd) to track the origin of Ruditapes philippinarum (clam) shells, and the results showed shell Ɛ_Nd_ is a powerful tool to discriminate the geographic location of clams. Studies have reported the application of terahertz (THz) spectroscopy [30], energy dispersive X-ray fluorescence spectrometry and near-infrared reflectance spectroscopy [31], multi-elemental analysis by energy dispersion X-ray fluorescence spectrometry combined with multivariate statistical analysis to discriminate the geographical origin of soybeans [32], and Chinese soybean paste characterization based on flavor profiles using HS-SPME-GC/MS, E-nose, and E-tongue combined with chemometrics [33]. The geographical origin of commercial soybeans sold in Vietnam and Argentina was explored using ICP-MS [34,35], and stable carbon and nitrogen isotopic compositions of soybeans from various cultivated regions of China were determined [36].

To date, there are no studies that have generated an isotope or elemental database suitable for verifying the geographical origin of domestic and imported soybeans into China. The objective of this study was to examine the chemometric discrimination ability for the geographical origin of soybeans using stable isotopes (*δ*^2^H, *δ*^18^O, *δ*^15^N, *δ*^13^C, and *δ*^34^S), non-metallic element content (% N, % C, and % S) and elemental profiles. The fingerprint information acquired by Isotope Ratio Mass Spectrometry (IRMS) and Inductively Coupled Plasma Mass Spectrometry (ICP-MS) were combined with multivariate statistical techniques such as PCA, LDA, and ANN to distinguish the geographical origin of four origins, including domestic and imported soybeans. We identified individual isotope trends and undertook multi-variate analyses to establish the most useful discriminatory chemometric models for country of origin verification. This study proposes a combined approach to trace the geographical origin of soybeans in order to solve the phenomenon of mislabeled countries of origin in Chinese markets and improve traceability efficiency in the event of fraud or food safety concerns.

## 2. Materials and Methods

### 2.1. Sample Collection and Preparation

All soybean samples were collected in 2020 from ports such as Shenzhen Port, Zhangjiakou Port, and others that are primarily involved in importing to China and analyzed by Shenzhen Customs, which resulted in the collection of 156 soybean samples from 4 countries: Brazil (*n* = 90), China (*n* = 33), USA (*n* = 6), and Argentina (*n* = 27). Soybean samples were dried to facilitate storage and transportation. The specific sampling information is shown in Table 1. Over 1 kg of soybeans were acquired for each sample, and samples were stored at room temperature (24 to 28 °C) in the laboratory until analysis. Soybean samples were washed with deionized water (Milli-Q water system, Darmstadt, Germany), dried again in an oven at 55 °C for 24 h to a constant weight, then ground to a fine powder (<80 μm) using a grinding mill (MM400, Retsch, German). Samples were stored in a desiccator for further stable isotopes and elemental profile analysis.

### 2.2. Stable Isotope Analysis

*δ*^2^H, *δ*^18^O, *δ*^15^N, *δ*^13^C, and *δ*^34^S values and N, C, and S elemental contents of soybean samples were measured using an Isotope Ratio Mass Spectrometer (IRMS) (DELTA V, Thermo Scientific, Bremen, Germany) equipped with an Elemental Analyzer (HT2000) (EA-IRMS, DELTA V advantage, Thermo Fisher, Bremen, Bremen (state), Germany), according to previously described methods [23,37].

Around 3 mg of each powdered soybean sample was weighed in duplicate into a 4 × 6 mm tin capsule for the simultaneous determination of *δ*^15^N, *δ*^13^C, *δ*^34^S and N, C, S content using a micro-balance (P6, Mettler, Switzerland) and tightly wrapped. The powdered samples were placed in an autosampler and converted to gases (N_2_, CO_2_, and SO_2_) in the EA. Evolved gases were passed through a drying tube containing Mg(ClO_4_)_2_. The EA combustion parameters were as follows: reaction oven: 1020 °C, column temperature: 70 °C, O_2_ flow: 175 mL/min, oxygen injection time: 3 s; carrier flow (helium gas): 200 mL/min.

Around 0.2 mg of each powdered soybean sample was weighed in triplicate into a 3.5 × 5 mm silver capsule for the simultaneous determination of *δ*^2^H and *δ*^18^O. Soybean samples and reference materials were exposed to local atmospheric conditions to equilibrate exchangeable water in the laboratory for at least 48 h before analysis. TC/EA parameters were as follows: furnace temperature: 1350 °C, column temperature: 90 °C, carrier flow (Helium gas): 110 mL/min.

The isotope ratio is denoted in delta notation (*δ*) in accordance with the following formula:

(1) δ(E i/j)=δE i/j=R i/jP−R i/jRefR i/jRef

where *i* and *j* denote the highest and the lowest atomic mass number of element *E*, respectively. *R_P_* and *R_Ref_* represent the ratio of the heavier and the lighter element (^13^C/^12^C, ^15^N/^14^N, ^34^S/^32^S, ^2^H/^1^H, and ^18^O/^16^O) of the sample and reference materials, respectively [38]. The delta values applied to the International System of Units (SI) were expressed in units of “per mil” (‰) or “miliurey” (mUr). In this study, we choose “‰” as the isotope values unit.

The *δ*^13^C values were expressed as relative to Vienna-Pee Dee Belemnite (V-PDB), *δ*^15^N to the air (air) scale, *δ*^34^S to Vienna-Canyon Diablo Troilite (V-CDT) scale, while *δ*^2^H and *δ*^18^O were expressed relative to the Vienna-Standard Mean Ocean Water (V-SMOW) scale. The isotope values of soybean samples were calculated against international standard reference materials as follows: USGS43 (Indian human hair powder, *δ*^13^C_V-DPB_ = −21.28‰, *δ*^15^N_Air_ = 8.44‰, *δ*^34^S_V-CDT_ = 10.46‰) was used for *δ*^13^C, *δ*^15^N and *δ*^34^S calibration, Sulfadiazine (C = 41.81%, N = 16.25%, S = 18.62%) was used as the quality control sample for elemental content of carbon, nitrogen, and sulfur (C %, N % and S %). USGS43 (Indian human hair powder, *δ*^2^H_V-SMOW_ = −50.3‰, *δ*^18^O_V-SMOW_ = +14.11‰), USGS54 (Canadian lodgepole pine wood powder, *δ*^2^H_V-SMOW_ = −150.4‰, *δ*^18^O_V-SMOW_ = +17.79‰), USGS56 (South African red ivory wood powder, *δ*^2^H_V-SMOW_ = −44‰, *δ*^18^O_V-SMOW_ = +27.23‰) were used for the *δ*^2^H and *δ*^18^O calibrations. Blanks were used, and standards were inserted for drift correction after every eight samples. The accuracy of carbon, nitrogen, and sulfur stable isotopes is ±0.1‰, ±0.2‰, and ±0.4‰, respectively. The accuracy of hydrogen and oxygen stable isotopes was ±1.2‰ and ±0.3‰, respectively.

### 2.3. Elemental Analysis

A total of 23 macro, micro, and trace elements (Na, Mg, Al, K, Ca, V, Cr, Mn, Fe, Co, Ni, Cu, Zn, Ga, As, Se, Rb, Sr, Cd, Cs, Ba, Tl, Pb) were quantified using Inductively Coupled Plasma Mass Spectrometry (ICP-MS, Agilent, 8800, Agilent Technologies, Tokyo, Japan). Around 0.5 g of powdered soybean was weighed and digested using a microwave digestion system. Samples were digested in Teflon digestion vessels containing a mixture of 6.0 mL of HNO_3_ (AR, 65%, *w*/*w*, Merck, Darmstadt, Germany) and 2.0 mL of H_2_O_2_ (AR, 20%, *w*/*w*, Merck, Darmstadt, Germany, Merck) for 2 h. The digestion temperature program was as follows: from 0 °C to 100 °C in 10 min, increasing to 180 °C in 15 min, and increasing to 240 °C in 15 min before being kept at 240 °C for 10 min. After mineralization, the obtained extracts were redissolved to a total volume of 25 mL with double Milli-Q water (MΩ > 18.2 M) and subsequently analyzed by ICP-MS. A multi-element standard solution (ICP-MS-CAL2-1, AccuStandard, Thermo Fisher, Bremen, Germany) in 0.5% nitric acid (chromatographically pure) was used to determine the sensitivity factors of all elements over the entire mass range measured by semi-quantitative mode dilution samples.

### 2.4. Statistical Analysis

Data processing was accomplished using SPSS 24.0 for macOS (IBM, Armonk, New York, NY, USA), and the results were expressed as the mean value ± standard deviation. GraphPad Prism 8.4.0 for macOS (GraphPad Software Inc., San Diego, CA, USA) was used to produce the figures. One-way analysis of variance (ANOVA) followed by the post hoc Tukey’s HSD was used to evaluate the differences in the samples with the isotope and elemental variations from the four countries, respectively. This study considered a statistically significant difference occurred at a *p*-value ≤ 0.05. Principal component analysis (PCA) was used in the analysis process to reduce the dimensionality of the dataset and describe the variability of the system with fewer variables. Linear discriminant analysis (LDA) was used to find the linear combinations of noted attributes that can clearly separate two or more classes of objects [39]. Backpropagation artificial neural networks (BP-ANN) can learn and store a large number of input–output pattern maps. It is a kind of mathematical equation that does not need to reveal the mapping in advance. PCA, LDA, and BP-ANN analysis were implemented in SPSS 24.0 for macOS (IBM, Armonk, New York, NY, USA).

## 3. Results and Discussion

### 3.1. Isotopes and Non-Metallic Elements of Soybeans from Different Geographical Origins

**Figure 1:**
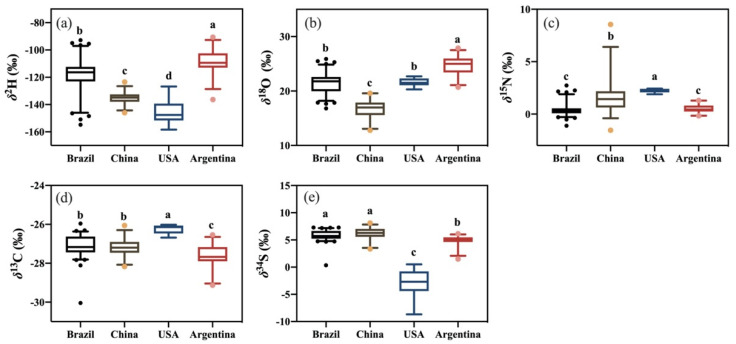
Box–Whisker plots comparing four geographical origins for (**a**) *δ*^2^H, (**b**) *δ*^18^O, (**c**) *δ*^15^N, (**d**) *δ*^13^C, and (**e**) *δ*^34^S compositions. Box plots display the lower, median, and upper quartile values, using vertical lines to show the range from the 10 to 90 percentiles. Outliers are depicted as dots. Different lowercase letters indicate that significant differences were recorded in the soybeans from the four origins (*p* < 0.05).

**Figure 2:**
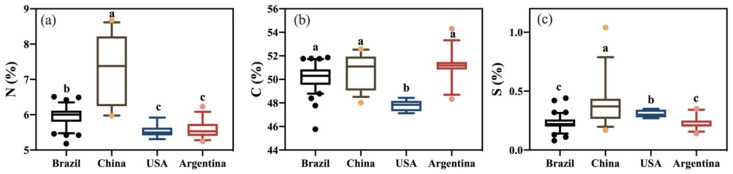
Box–Whisker plots of (**a**) % N, (**b**) % C, (**c**) % S of four geographical origins. Box plots display the lower, median, and upper quartile values, using vertical lines to show the range from the 10 to 90 percentiles. Outliers are depicted as dots. Different lowercase letters indicate that significant differences were recorded in the soybeans from the four origins (*p* < 0.05).

Stable isotope and elemental profiles of imported and Chinese domestic soybean samples were analyzed and summarized by mean value ± standard deviation in Table 2. Box–Whisker plots of stable isotopes and non-metallic element contents are shown in Figure 1 and Figure 2. Analysis of bulk stable isotope and non-metallic elements using ANOVA showed there were significant differences (*p* < 0.05) among eight variables from the four origins (Brazil, China, the USA, and Argentina). The *δ*^2^H, *δ*^18^O, *δ*^15^N, *δ*^13^C, and *δ*^34^S values ranged from −158.4‰ to −90.6‰, 12.8‰ to 27.9‰, −1.5‰ to 8.6‰, −30.0‰ to −26.0‰, and −8.7‰ to 8.1‰, respectively. Average *δ*^2^H, *δ*^18^O, *δ*^15^N, *δ*^13^C, and *δ*^34^S values were −121.9‰, 21.0‰, 0.7‰, −27.2‰, and 5.4‰, respectively. 

Soybean *δ*^13^C values were within the expected range for C_3_ plants, as *δ*^13^C values of all soybean samples ranged from −30.0‰ to −26.0‰. Mean *δ*^13^C values of soybean samples from the USA (−26.2 ± 0.3‰) were slightly more positive than other origins, and *δ*^13^C values of soybean samples from Argentina had the most negative mean *δ*^13^C values (−27.6 ± 0.6‰). Some environmental factors can affect the *δ*^13^C values of plants, such as drought, solar radiation intensity, low temperature, low pressure, and ozone stress [40]. Previous studies proved that under the influence of temperature, light intensity, and humidity, the δ^13^C values of plants in humid cultivation areas were more negative [21]. Argentina and Brazil are located in South America and receive abundant rainfall, with most areas receiving more than 1000 mm of precipitation per year, making it the wettest continent in the world. Conversely, USA soybeans are grown at higher latitudes with a cooler climate, which reduces stomatal conductance during photosynthesis and discriminates against ^13^C.

The average of δ^15^N values of Chinese soybeans (1.6 ± 1.7‰) was consistent with previously reported results [36,41]. The maximum mean *δ*^15^N value of soybeans was 2.2 ± 0.2‰ from the USA, and the minimum mean *δ*^15^N value was found in Brazilian and Argentinian soybeans with an average of 0.4‰ and 0.5%. The use of nitrogen fertilizer is generally not recommended for legumes because, under favorable conditions, legumes are able to grow well from existing soil N plus N_2_ derived from symbiotic nitrogen fixation [42]. In order to minimize soybean production costs, high natural soil fertility is particularly important to ensure good soybean quality. The *δ*^15^N value of soybeans from Brazil and Argentina is close to that of atmospheric N_2_ (0‰ by definition), and it is interpreted that these values result from the fractionation of the legume–Rhizobium symbiosis during biological nitrogen fixation (BNF), which is generally small. This results in the *δ*^15^N value of the legume approaching zero when there is high symbiotic dependence. [13,43]. Moreover, the depleted nitrogen isotopic composition of soybeans in Brazil and Argentina suggests the utilization of synthetic fertilizers in their cultivation. USA soybeans are typically grown in deep “corn belt” soils where the soil is more fertile [44]. The natural climate and environmental conditions are suitable for soybean cultivation. Most USA soybean-producing areas have low and flat terrain. Fertile black grassland soil and chernozem soil are widely distributed in the USA, resulting in the highest mean *δ*^15^N values of soybeans of the four studied origins. *δ*^15^N values typically rely on agricultural practices, such as the type of fertilizer applied (organic or synthetic) or agricultural management. Soybeans cultivated in China exhibit the largest *δ*^15^N standard deviation, potentially due to the different cultivation methods across China. It is possible that certain Chinese soybean samples have been cultivated using organic farming instead of conventional practices. Nonetheless, this hypothesis could not be substantiated due to the unavailability of soil and fertilizer data.

*δ*^34^S values of plants do not clearly distinguish production methods (conventional versus organic) or fertilizer type but are generally correlated with local geological and soil conditions [13,45]. Chinese and Brazilian soybeans had the highest mean *δ*^34^S values (6.1‰ and 5.9‰), while the USA had the lowest mean *δ*^34^S values (−3.0‰). The δ^34^S values of USA soybeans had the widest isotopic distribution range compared to other origins, possibly due to a wider range of different geological sulfur sources and cultivation environments found in the Midwestern states of Iowa, Illinois, Minnesota, and Indiana (order of top producing states) [46].

**Figure 3:**
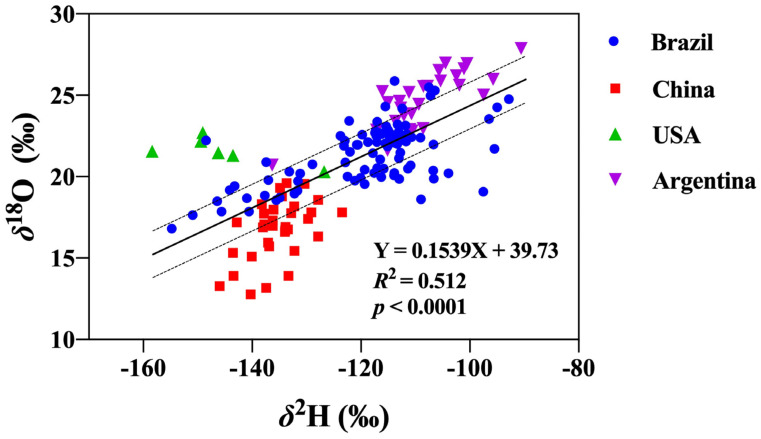
Comparison of *δ*^2^H and *δ*^18^O values in soybean samples from four origins (*p* < 0.0001).

Soybean *δ*^2^H and *δ*^18^O values were significantly different among the four origins. Argentinean soybean had the most positive *δ*^2^H and *δ*^18^O values (−108.6‰ and 24.7‰, respectively). The lowest mean soybean *δ*^2^H value was found in the USA (−145.8‰), and the lowest mean soybean *δ*^18^O value was found in China (16.7‰). The climate of Argentina’s agricultural regions is more tropical (humid and warm) than other countries. In general, geographical temperature fractionation effects exist, where the concentration of ^18^O decreases with increasing latitude and cooler temperatures [23]. However, due to the lack of information about precise sampling locations, the effects of altitude and latitude are not clear in this study. Results suggest that Argentinian soybeans may be produced at lower altitudes or in warmer regions, closer to the coast than the other origins. Lower *δ*^2^H and *δ*^18^O values for Chinese soybeans suggest they are grown inland and potentially in cooler regions than the other countries. A comparison between *δ*^2^H and *δ*^18^O values in soybean samples from four origins is shown in Figure 3. Overall, *δ*^2^H and *δ*^18^O values of soybeans show a reasonable overall linear relationship (*R*^2^ = 0.512), although soybean isotope values cluster into their respective origins. 

Elemental nitrogen, carbon, and sulfur (% N, % C, and % S) of imported and domestic soybean samples ranged from 5.2% to 8.7%, 45.8% to 54.3%, and 0.1% to 1.0%, respectively. Average % N, % C, and % S contents were 6.2%, 50.4%, and 0.3%, respectively. A significant difference in N, C, and S elemental compositions of soybean from four origins was seen (*p* < 0.05). USA soybeans had the lowest N, C, and S element contents among the four producing countries, while Chinese soybeans had the highest elemental contents. Some soybean samples from the USA may have originated from the northern states, which have cooler temperatures and less sunshine, resulting in lower nitrogen, carbon, and sulfur storage in plant tissues. Significant elemental differences in soybeans found in the four origins indicate that these elemental contents may contribute towards distinguishing geographical origin.

### 3.2. Mineral Element Compositions of Soybeans from Different Geographical Origins

**Figure 4:**
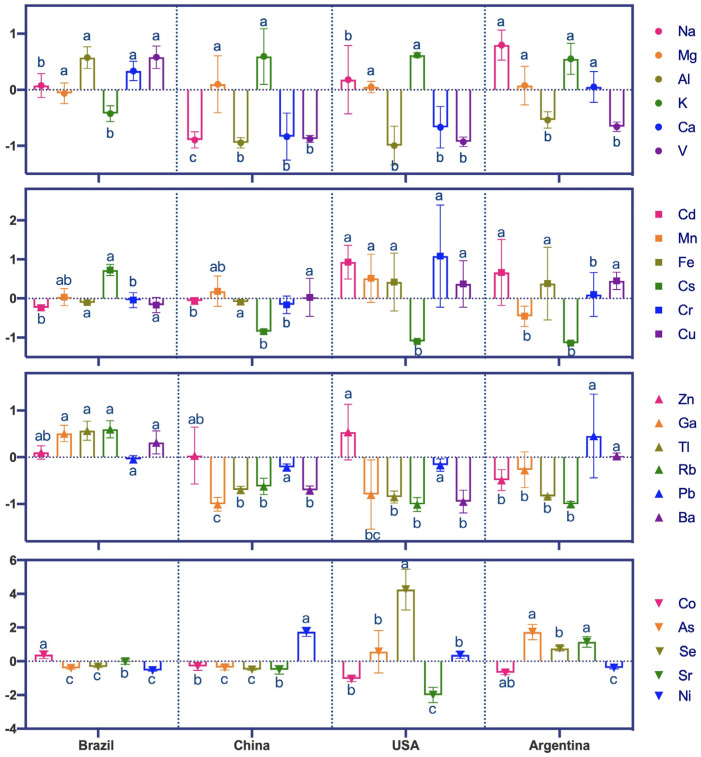
Histogram plots showing differences in 23 mineral compositions across four geographical origins. The concentrate of 23 mineral elements has been standardized in order to facilitate plotting, and the original results are shown in Table 2. Different lowercase letters indicate significant differences were recorded in the soybeans from the four origins (*p* < 0.05).

Mineral element abundance is shown in Table 2. The relative elemental concentrations were quite variable among soybeans from different countries but comparable with elements found in USA and Brazilian soybeans from previous studies [34]. The concentration range of most mineral elements from the four origins overlapped. Statistically significant differences (*p* < 0.05, same below) linked to the geographical origin of the samples were found for all 23 elements except Mg, Fe, Cu, and Pb. Soybean Mg and Cu contents were found to be effective origin indicators of Chinese and Japanese soybeans [47], but these elements were not considered important origin discriminators in this study, possibly due to the different soybean origins that were chosen. The most abundant soybean elements found in this study were K, Ca, and Mg. Soybean K content ranged between 314,785.98 μg/kg and 564,631.18 μg/kg, with an average value of 413,278.10 μg/kg. Histograms were made by standardizing each mineral element variable with its Z-score (Figure 4) in order to distinguish composition differences among the four production origins.

Elemental contents of Al, V, Co, Ga, Rb, Cs, and Tl were significantly higher in Brazilian soybeans than in the other three origins. Brazilian soybeans had the lowest concentrations of K, and Brazilian and Chinese soybeans had lower concentrations of AS, Se, and Cd than USA and Argentinean soybeans. Brazilian and Argentinean soybeans had lower concentrations of Ni. Chinese soybeans had significantly higher concentrations of Ni and the lowest concentration of Na. The elemental contents of Cr, Mn, Zn, and Se were significantly higher in USA soybeans than in other origins. Soybeans cultivated in Argentina had the highest concentrations of Na, As, and Sr and the lowest concentrations of Mn. Significant differences in mineral element contents are attributed to different geological and environmental conditions, climatic uptake factors, and human interventions from each origin. 

Soybean production in Brazil is concentrated in the “Cerrado” region, a prairie-like plain with less fertile soils that are rich in aluminum, highly acidic, and lack phosphorus and nitrogen [44]. The low soil pH found in Brazil increases the bioavailability, and consequently the plant uptake, of aluminum and iron [48] relative to other origins.

### 3.3. Soybean Origin Model Evaluation and Classification Using Chemometrics

Chemometrics is an interdisciplinary method that employs multivariate statistics to extract essential information from intricate chemical systems and can now discern subtle differences in measurements among target products [49]. In this study, we applied unsupervised and supervised pattern recognition methods, including PCA, LDA, and BP-ANN, to analyze soybean data.

#### 3.3.1. Principal Component Analysis (PCA)

**Figure 5:**
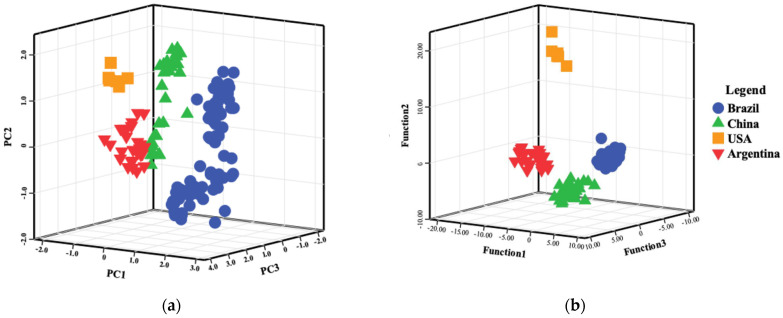
3D-Ordinations of multivariate statistical analyses using a combined approach of stable isotopes, three non-metallic element contents, and 23 element compositions for four origins showing (**a**) principal component analysis (PCA) and (**b**) linear discriminant analysis (LDA). Each symbol type represents an individual origin.

Unsupervised PCA was performed using a combination of stable isotopes and non-metallic and mineral element contents. Overall, PCA confirmed the ability of isotopes and elements to provide unsupervised origin classification and discriminate the soybeans based on origin. The first six principal components (PCs) in the PCA model accounted for 80.49% of the total variance of the original variables, and the first three PCs explained 58.05% of the total variance of the original variables. Meanwhile, a 3D-Ordinations plot, constructed using the first three PCs, showed that soybean samples from four origins were generally well clustered without overlap (Figure 5a). PCA was shown to be a useful technique to initially determine the geographical origin of soybeans, but due to the lower total variance of the variables, other models were investigated. 

#### 3.3.2. Linear Discriminant Analysis (LDA) and BP–Artificial Neural Network (BP–ANN)

More meaningful classification models were constructed using LDA and BP-ANN in combination with stable isotope and element data. In order to establish a stronger origin classification model, identify effective classification variables, and better classify soybean samples from different origins, supervised linear discriminant analysis (LDA) and unsupervised BP artificial neural network analysis (BP-ANN) were performed using both stable isotopes and multi-element contents.

LDA analysis of soybean samples from four origins revealed discriminant accuracies of 85.3% for stable isotopes only, 75.0% for non-metallic elemental contents only, and 100.0% for mineral elemental compositions only (Table 3 A–C). The discrimination accuracy for mineral elements of soybeans was higher than stable isotopes and non-metallic elements and was able to completely separate soybeans from the four origins. However, a single USA soybean sample was mis-classified as Argentinian, resulting in an accuracy rate of 99.4% in cross-validation. LDA results show that Brazilian soybeans had the lowest discriminant accuracy when using stable isotope variables only, and most of the mis-classified Brazilian soybeans were classed as Argentinian. This higher rate of misclassification using stable isotopes may be caused by a closer geographical proximity between Argentina and Brazil than the other two origins, and these two producing origins may share similar climate and farming conditions. In order to obtain a higher discriminant rate and more accurately distinguish the four soybean-producing origins, the three groups of variables were combined into a single linear discriminant analysis model. The results showed that a combined stable isotope and multi-element approach could completely distinguish soybeans from all origins, and the original accuracy and cross-validation accuracy were both up to 100% (Table 3 D).

**Figure 6:**
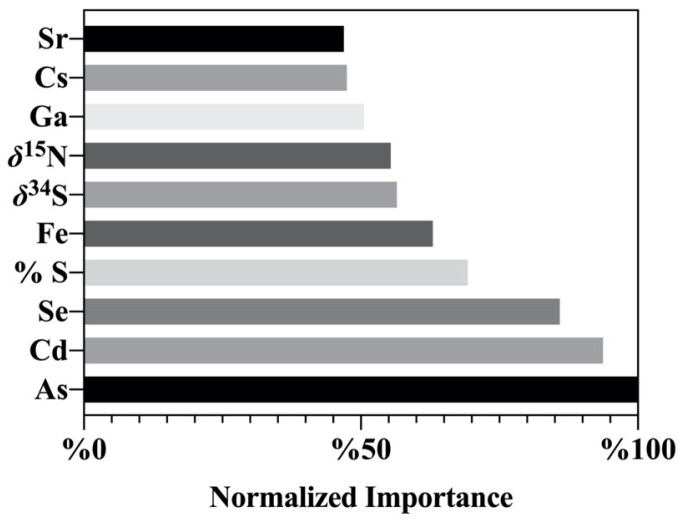
Stable isotopes and elemental composition variable importance for the contribution towards origin verification using an ANN classification method.

A BP-ANN model was built with 70% training samples and 30% testing samples. Training and testing samples from each origin were selected randomly. The normalized importance for each elemental and stable isotope variable for the contribution to origin verification is shown in Figure 6. As, Cd, Se, % S, and Fe were identified as the top five important variables contributing to origin verification, and they all showed a strong ability to identify the origin of Chinese and imported soybeans. The ANN model was able to discriminate soybeans from all four origins, correctly identifying all durian origins with up to 100% training accuracy and 100% testing accuracy (Table 3 E). Classification accuracies of the BP-ANN model were found to be similar to the LDA model where the mineral element model provided the best soybean origin, although a combined approach with all variables completely distinguished soybeans from all origins.

While successfully differentiating soybeans from four origins using stable isotopes and mineral elements combined with chemometrics, it is crucial to acknowledge that bulk stable isotope values and mineral elements represent overall averages of components. In specific cases, these values may not fully capture isotopic and elemental variations among different samples. Recently, there has been increasing attention paid to Compound-Specific Stable Isotope Analysis (CSIA) due to its ability to provide unique information about food components at the molecular level, reflecting geographical, cultivation, and natural ecosystem processes [50]. Our forthcoming strategy aims to enhance origin discrimination by utilizing CSIA to discern isotopic variations among specific components (e.g., amino acids, fatty acids) in soybeans from different geographical origins.

## 4. Conclusions

Stable isotope and elemental compositions were used to develop an origin identification model to determine the geographical origin of soybeans from four countries. The model could be further used to improve the origin traceability of similar leguminous food products. Chinese soybeans and imported soybeans were characterized by chemometric exploratory techniques and classification parameters such as PCA, LDA, and ANN using stable isotopes (*δ*^2^H, *δ*^18^O, *δ*^15^N, *δ*^13^C, and *δ*^34^S), non-metallic element contents (% N, % C, and % S) and 23 mineral elements to verify their geographical origins. PCA results showed the four groups of soybean samples were distributed independently. A combined stable isotope, non-metallic, and mineral element contents data fusion approach combined with LDA afforded a more accurate soybean origin classification (with discriminant accuracy rates of 100% for both the testing and training sets) than a single analytical approach. Likewise, BP-ANN gave a 100% accuracy rate for both testing and training sets of four origin groups, and As, Cd, Se, % S, and Fe were identified as the top five important variables. An accurate origin identification tool using isotope and elemental fingerprints combined with chemometrics could avoid any potential conflict of interest arising from incorrect origin identification among soybeans. Regulatory uptake and use of soybean geographical origin models will prevent fraud or mislabeling at a global market level and could contribute to fairer international trading.

## Figures and Tables

**Table 1:** Soybean sample information.

Origin	Proportion of Total Imports % ^1^					
	2015	2016	2017	2018	2019	2020
Brazil	49.09	45.7	53.31	75.1	65.11	64.07
China	-	-	-	-	-	-
USA	34.76	40.44	34.39	18.9	19.13	25.8
Argentina	11.55	9.63	6.89	1.66	9.92	7.43
Total	87.61	90.95	88.52	80.92	93.1	97.3

^1^ Data from unpublished China customs yearbooks.

**Table 2:** One-way analysis of variance (ANOVA) of soybeans from four geographical origins using stable isotope and elemental contents. Element contents from ICP-MS are given in μg/kg (where 1 μg/kg = 1 ppb). The values in the table are expressed as mean ± standard deviation.

Isotope/Element	Brazil (*n* = 90)	China (*n* = 33)	USA (*n* = 6)	Argentina (*n* = 27)
*δ*^2^H (‰)	−119.30 ± 12.77 ^b^	−135.31 ± 4.93 ^c^	−145.57 ± 10.47 ^d^	−108.68 ± 8.62 ^a^
*δ*^18^O (‰)	21.37 ± 1.90 ^b^	16.73 ± 1.83 ^c^	21.58 ± 0.82 ^b^	24.73 ± 1.73 ^a^
*δ*^15^N (‰)	0.37 ± 0.57 ^c^	1.59 ± 1.68 ^b^	2.24 ± 0.21 ^a^	0.51 ± 0.41 ^c^
*δ*^13^C (‰)	−27.10 ± 0.56 ^b^	−27.18 ± 0.49 ^b^	−26.24 ± 0.25 ^a^	−27.63 ± 0.60 ^c^
*δ*^34^S (‰)	5.86 ± 0.97 ^a^	6.13 ± 1.14 ^a^	−2.96 ± 3.09 ^c^	4.90 ± 0.93 ^b^
% N (%)	5.96 ± 0.25 ^b^	7.32 ± 0.96 ^a^	5.54 ± 0.21 ^c^	5.57 ± 0.22 ^c^
% C (%)	50.22 ± 0.97 ^a^	50.63 ± 1.44 ^a^	47.79 ± 0.5 ^b^	51 ± 1.10 ^a^
% S (%)	0.23 ± 0.06 ^c^	0.38 ± 0.16 ^a^	0.31 ± 0.03 ^b^	0.23 ± 0.05 ^c^
Na	291.92 ± 130.33 ^b^	167.32 ± 52.63 ^c^	305.2 ± 74.69 ^b^	384.53 ± 87.04 ^a^
Mg	47,475.94 ± 5428.06 ^a^	48,475.48 ± 8888.79 ^a^	48,173.5 ± 609.46 ^a^	48,327.81 ± 5394.10 ^a^
Al	495.87 ± 226.27 ^a^	122.71 ± 64.57 ^b^	110.41 ± 80.97 ^b^	223.00 ± 91.24 ^b^
K	386,682.09 ± 42,613.11 ^b^	450,478.54 ± 87,922.42 ^a^	452,010.02 ± 2680.36 ^a^	447,857.14 ± 43,826.06 ^a^
Ca	10,216.69 ± 1272.25 ^a^	8419.93 ± 1818.33 ^b^	8676.26 ± 541.98 ^b^	9780.58 ± 1063.6 ^a^
V	1.03 ± 0.55 ^a^	0.19 ± 0.10 ^b^	0.16 ± 0.05 ^b^	0.31 ± 0.12 ^b^
Cr	2.07 ± 1.69 ^b^	1.85 ± 1.16 ^b^	4.16 ± 2.31 ^a^	2.34 ± 2.65 ^b^
Mn	684.92 ± 141.78 ^ab^	705.50 ± 150.02 ^ab^	750.78 ± 80.31 ^a^	617.89 ± 90.26 ^b^
Fe	1606.96 ± 220.29 ^a^	1637.96 ± 386.43 ^a^	2597.49 ± 1321.92 ^a^	2522.00 ± 4400.61 ^a^
Co	4.02 ± 1.77 ^a^	2.88 ± 1.10 ^b^	1.65 ± 0.24 ^c^	2.25 ± 0.45 ^bc^
Ni	37.29 ± 17.62 ^c^	242.28 ± 68.19 ^a^	119.35 ± 16.85 ^b^	50.80 ± 11.49 ^c^
Cu	220.69 ± 35.80 ^a^	228.24 ± 52.77 ^a^	241.64 ± 21.80 ^a^	244.6 ± 21.63 ^a^
Zn	799.96 ± 74.03 ^ab^	792.94 ± 185.04 ^ab^	847.03 ± 61.09 ^a^	736.31 ± 60.58 ^b^
Ga	0.15 ± 0.07 ^a^	0.03 ± 0.03 ^c^	0.05 ± 0.06 ^bc^	0.09 ± 0.08 ^b^
As	0.18 ± 0.05 ^c^	0.19 ± 0.08 ^c^	0.36 ± 0.22 ^b^	0.58 ± 0.21 ^a^
Se	1.06 ± 0.81 ^c^	0.35 ± 0.47 ^c^	20.46 ± 4.88 ^a^	5.66 ± 1.46 ^b^
Rb	516.41 ± 153.42 ^a^	301.93 ± 86.00 ^b^	233.86 ± 24.65 ^b^	235.15 ± 25.19 ^b^
Sr	227.50 ± 45.78 ^b^	198.62 ± 43.47 ^b^	109.10 ± 26.05 ^c^	297.76 ± 48.43 ^a^
Cd	0.38 ± 0.45 ^b^	0.60 ± 0.30 ^b^	1.83 ± 0.51 ^a^	1.50 ± 2.64 ^a^
Cs	3.24 ± 1.12 ^a^	0.61 ± 0.28 ^b^	0.20 ± 0.01 ^b^	0.12 ± 0.03 ^b^
Ba	301.84 ± 195.87 ^a^	130.17 ± 41.97 ^b^	89.35 ± 39.22 ^b^	253.21 ± 26.89 ^a^
Tl	0.09 ± 0.06 ^a^	0.02 ± 0.01 ^b^	0.01 ± 0.01 ^b^	0.01 ± 0 ^b^
Pb	0.38 ± 0.30 ^a^	0.25 ± 0.15 ^a^	0.29 ± 0.10 ^a^	0.78 ± 1.81 ^a^

^a–d^ Different superscript letter values indicate a significant difference among groups (*p* < 0.05).

**Table 3:** Variability in discriminatory accuracy for soybean origin classification based on stable isotope and elemental compositions using LDA and BP-ANN models with diverse parameters.

Model	Geographical Origin	Predicted Group (Original/Cross-Validated)				Correctly Classified % (Original/Cross-Validated)
		Brazil	China	USA	Argentina	
LDA	A. Stable isotopes only					
	Brazil (*n* = 90)	70/69	7/8	0/0	13/13	77.8/76.7
	China (*n* = 33)	2/2	31/31	0/0	0/0	93.9/93.9
	USA (*n* = 6)	0/0	0/0	6/6	0/0	100.0/100.0
	Argentina (*n* = 27)	1/2	0/0	0/0	26/25	96.3/92.6
					total	85.3/84.0
	B. Non-metallic elemental contents only					
	Brazil (*n* = 90)	66/65	0/0	10/10	14/15	73.3/72.2
	China (*n* = 33)	5/5	22/22	6/6	0/0	66.7/66.7
	USA (*n* = 6)	0/0	0/0	6/6	0/0	100.0/100.0
	Argentina (*n* = 27)	3/3	0/0	1/1	23/23	85.2/85.2
					total	75.0/74.4
	C. Mineral elemental compositions only					
	Brazil (*n* = 90)	90/90	0/0	0/0	0/0	100.0/100.0
	China (*n* = 33)	0/0	33/33	0/0	0/0	100.0/100.0
	USA (*n* = 6)	0/0	0/0	6/5	0/1	100.0/83.3
	Argentina (*n* = 27)	0/0	0/0	0/0	27/27	100.0/100.0
					total	100.0/99.4
	D. Composite indicator					
	Brazil (*n* = 90)	90/90	0/0	0/0	0/0	100.0/100.0
	China (*n* = 33)	0/0	33/33	0/0	0/0	100.0/100.0
	USA (*n* = 6)	0/0	0/0	6/6	0/0	100.0/100.0
	Argentina (*n* = 27)	0/0	0/0	0/0	27/27	100.0/100.0
					total	100.0/100.0
BP-ANN	E. Composite indicator (N = Training/Testing)					
	Brazil (*n* = 90)	58/32	0/0	0/0	0/0	100.0/100.0
	China (*n* = 33)	0/0	24/9	0/0	0/0	100.0/100.0
	USA (*n* = 6)	0/0	0/0	3/3	0/0	100.0/100.0
	Argentina (*n* = 27)	0/0	0/0	0/0	23/4	100.0/100.0
					total	100.0/100.0

## Data Availability

The data used to support the findings of this study can be made available by the corresponding author upon request.

## References

[1] Zhang M., Liu S., Wang Z., Yuan Y., Zhang Z., Liang Q., Yang X., Duan Z., Liu Y., Kong F. (2022). Progress in soybean functional genomics over the past decade. Plant Biotechnol. J..

[2] Shea Z., Singer W.M., Zhang B. (2020). Soybean production, versatility, and improvement. Legume Crops-Prospects, Production and Uses.

[3] Wijewardana C., Reddy K.R., Bellaloui N. (2019). Soybean seed physiology, quality, and chemical composition under soil moisture stress. Food Chem..

[4] Hartman G.L., West E.D., Herman T.K. (2011). Crops that feed the World 2. Soybean—Worldwide production, use, and constraints caused by pathogens and pests. Food Secur..

[5] Statistics. www.stats.gov.cn.

[6] Thakur M., Hurburgh C.R. (2007). Quality of US Soybean Meal Compared to the Quality of Soybean Meal from Other Origins. J. Am. Oil Chem. Soc..

[7] Stojković L., Elbilali H., Berjan S., Despotovic A., Pospisil M. Protected Geographical Indications as a tool for valorising traditional and typical agro-food products and improving rural livelihoods in Serbia: Case of “Pirotski kachkaval” cheese from Stara Planina region. Proceedings of the International Symposium on Agriculture.

[8] Commission E. (1992). Council Regulation (EEC) No 2081/92 of 14 July 1992 on the protection of geographical indications and designations of origin for agricultural products and foodstuffs. J. Eur. Union.

[9] Muñoz-Redondo J.M., Bertoldi D., Tonon A., Ziller L., Camin F., Moreno-Rojas J.M. (2022). Multi-element and stable isotopes characterization of commercial avocado fruit (*Persea americana* Mill) with origin authentication purposes. Food Control.

[10] Gale F., Valdes C., Ash M. (2019). Interdependence of China, United States, and Brazil in soybean trade. New York: US Department of Agriculture’s Economic Research Service (ERS) Report.

[11] Medina S., Pereira J.A., Silva P., Perestrelo R., Câmara J.S. (2019). Food fingerprints—A valuable tool to monitor food authenticity and safety. Food Chem..

[12] Brüggemann N., Gessler A., Kayler Z., Keel S., Badeck F., Barthel M., Boeckx P., Buchmann N., Brugnoli E., Esperschütz J. (2011). Carbon allocation and carbon isotope fluxes in the plant-soil-atmosphere continuum: A review. Biogeosciences.

[13] Inacio C.T., Chalk P.M., Magalhaes A.M. (2015). Principles and limitations of stable isotopes in differentiating organic and conventional foodstuffs: 1. Plant products. Crit. Rev. Food Sci. Nutr..

[14] Camin F., Perini M., Bontempo L., Fabroni S., Faedi W., Magnani S., Baruzzi G., Bonoli M., Tabilio M., Musmeci S. (2011). Potential isotopic and chemical markers for characterising organic fruits. Food Chem..

[15] Mizota C., Sasaki A. (1996). Sulfur isotope composition of soils and fertilizers: Differences between Northern and Southern hemispheres. Geoderma.

[16] Rogers K.M., Martin A.P., Pradel G., Yuan Y., Zhang Y., Turnbull R.E. (2022). Elemental and isotopic compositions of New Zealand regional soils identifies human and climate-induced effects. Appl. Geochem..

[17] Thomas J., Rose T. (2003). Environmental isotopes in hydrogeology. Environ. Geol..

[18] Nie J., Weng R., Li C., Liu X., Wang F., Rogers K.M., Qian Y., Zhang Y., Yuan Y. (2022). Chemometric origin classification of Chinese garlic using sulfur-containing compounds, assisted by stable isotopes and bioelements. Food Chem..

[19] Qi J., Li Y., Zhang C., Wang C., Wang J., Guo W., Wang S. (2020). Geographic origin discrimination of pork from different Chinese regions using mineral elements analysis assisted by machine learning techniques. Food Chem..

[20] Kelly S., Heaton K., Hoogewerff J. (2005). Tracing the geographical origin of food: The application of multi-element and multi-isotope analysis. Trends Food Sci. Technol..

[21] Wang Y., Kang L., Zhao Y., Xiong F., Yuan Y., Nie J., Huang L., Yang J. (2022). Stable isotope and multi-element profiling of Cassiae Semen tea combined with chemometrics for geographical discrimination. J. Food Compos. Anal..

[22] Ni X., Li X., Ran G., Chen J., Jiang X., Sun J., Bai W. (2022). Determination of the geographical origin of *Trachinotus ovatus* and *Pampus argenteus* in China by multi-element and stable isotope analysis. Food Chem..

[23] Zhou X., Wu H., Pan J., Chen H., Jin B., Yan Z., Xie L., Rogers K.M. (2021). Geographical traceability of south-east Asian durian: A chemometric study using stable isotopes and elemental compositions. J. Food Compos. Anal..

[24] Li C., Nie J., Zhang Y., Shao S., Liu Z., Rogers K.M., Zhang W., Yuan Y. (2022). Geographical origin modeling of Chinese rice using stable isotopes and trace elements. Food Control.

[25] Liu X., Zhao Y., Qi P., Liu Y., Li X., Deng W., Zhang J., Sadiq F.A., Sang Y., Zhang A. (2022). Origin verification of Chinese concentrated apple juice using stable isotopic and mineral elemental fingerprints coupled with chemometrics. J. Food Compos. Anal..

[26] Akamatsu F., Shimizu H., Hayashi S., Kamada A., Igi Y., Koyama K., Yamada O., Goto-Yamamoto N. (2022). Chemometric approaches for determining the geographical origin of Japanese Chardonnay wines using oxygen stable isotope and multi-element analyses. Food Chem..

[27] Wu H., Lin G., Tian L., Yan Z., Yi B., Bian X., Jin B., Xie L., Zhou H., Rogers K.M. (2020). Origin verification of French red wines using isotope and elemental analyses coupled with chemometrics. Food Chem..

[28] Del Rio-Lavín A., Weber J., Molkentin J., Jiménez E., Artetxe-Arrate I., Pardo M.Á. (2022). Stable isotope and trace element analysis for tracing the geographical origin of the *Mediterranean mussel* (*Mytilus galloprovincialis*) in food authentication. Food Control.

[29] Tanaka K., Zhao L., Tazoe H., Iizuka T., Murakami-Sugihara N., Toyama K., Yamamoto T., Yorisue T., Shirai K. (2022). Using neodymium isotope ratio in *Ruditapes philippinarum* shells for tracking the geographical origin. Food Chem.

[30] Wei X., Zhu S., Zhou S., Zheng W., Li S. (2020). Identification of Soybean Origin by Terahertz Spectroscopy and Chemometrics. IEEE Access.

[31] Kim N., Jang M., Jo J., Park J., Kim A., Hwang I. (2022). Application of energy dispersive X-ray fluorescence spectrometry and near-infrared reflectance spectroscopy combined with multivariate statistical analysis for discriminating the geographical origin of soybeans. Food Control.

[32] Lai H., Xi J., Sun J., He W., Spectroscopy A. (2020). Multi-elemental Analysis by Energy Dispersion X-ray Fluorescence Spectrometry and Its Application on the Traceability of Soybean Origin. At. Spectrosc..

[33] Yu S., Huang X., Wang L., Ren Y., Zhang X., Wang Y. (2022). Characterization of selected Chinese soybean paste based on flavor profiles using HS-SPME-GC/MS, E-nose and E-tongue combined with chemometrics. Food Chem..

[34] Nguyen-Quang T., Bui-Quang M., Truong-Ngoc M. (2021). Rapid Identification of Geographical Origin of Commercial Soybean Marketed in Vietnam by ICP-MS. J. Anal. Methods Chem..

[35] Hidalgo M.J., Fechner D.C., Ballabio D., Marchevsky E.J., Pellerano R.G. (2020). Traceability of soybeans produced in Argentina based on their trace element profiles. J. Chemom..

[36] Wu Y., Luo D., Dong H., Wan J., Luo H., Xian Y., Guo X., Qin F., Han W., Wang L. (2015). Geographical origin of cereal grains based on element analyser-stable isotope ratio mass spectrometry (EA-SIRMS). Food Chem..

[37] Zhou X., Yan Z., Jin B., Wu Y., Xie L., Chen H., Lin G., Zhao Y., Rogers K.M., Wu H. (2021). Origin verification of imported infant formula and fresh milk into China using stable isotope and elemental chemometrics. Food Control.

[38] Brand W.A., Coplen T.B., Vogl J., Rosner M., Prohaska T. (2014). Assessment of international reference materials for isotope-ratio analysis (IUPAC Technical Report). Pure Appl. Chem..

[39] Narayan Y. (2020). Hb vsEMG signal classification with time domain and Frequency domain features using LDA and ANN classifier. Mater. Today Proc..

[40] Yun S.I. (2008). Stable C and N Isotopes: A Tool to Interpret Interacting Environmental Stresses on Soil and Plant. J. Appl. Biol. Chem..

[41] Oh H.-J., Ko G.-A., Yang M.-Y., Kim Y.-J. (2021). Stable isotope ratio analysis for origin authentication of red pepper powders and soybeans. J. Korean Soc. Food Sci. Nutr..

[42] Hardarson G., Zapata F., Danso S. (1984). Effect of plant genotype and nitrogen fertilizer on symbiotic nitrogen fixation by soybean cultivars. Plant Soil.

[43] Rogers K.M. (2008). Nitrogen isotopes as a screening tool to determine the growing regimen of some organic and nonorganic supermarket produce from New Zealand. J. Agric. Food Chem..

[44] Huerta A.I., Martin M.A. Soybean Production Costs: An Analysis of the United States, Brazil, and Argentina. Proceedings of the 2002 Annual Meeting.

[45] Georgi M., Voerkelius S., Rossmann A., Gramann J., Schnitzler W.H. (2005). Multielement Isotope Ratios of Vegetables from Integrated and Organic Production. Plant Soil.

[46] Chang W.-S., Lee H.-I., Hungria M. (2015). Soybean Production in the Americas. Principles of Plant-Microbe Interactions.

[47] Otaka A., Hokura A., Nakai I. (2014). Determination of trace elements in soybean by X-ray fluorescence analysis and its application to identification of their production areas. Food Chem..

[48] Leibold K., Baumel C.P., Wisner R.N., Mcvey M.J. (2002). Brazil’s Crop Production System Holds Potential. Staff General Research Papers Archive.

[49] Soni K., Frew R., Kebede B. (2023). A review of conventional and rapid analytical techniques coupled with multivariate analysis for origin traceability of soybean. Crit. Rev. Food Sci. Nutr..

[50] Liu H., Nie J., Liu Y., Wadood S.A., Rogers K.M., Yuan Y., Gan R.-Y. (2023). A review of recent compound-specific isotope analysis studies applied to food authentication. Food Chem..

